# NECTAR: a database of codon-centric missense variant annotations

**DOI:** 10.1093/nar/gkt1245

**Published:** 2013-11-30

**Authors:** Sungsam Gong, James S. Ware, Roddy Walsh, Stuart A. Cook

**Affiliations:** ^1^NIHR Cardiovascular Biomedical Research Unit, Royal Brompton and Harefield NHS Foundation Trust and Imperial College London, London SW3 6NP, UK, ^2^National Heart and Lung Institute, Imperial College, London SW3 6LY, UK, ^3^National Heart Centre Singapore, Singapore 168752, Singapore and ^4^Cardiovascular & Metabolic Disorders, Duke National University of Singapore, Singapore 169857, Singapore

## Abstract

NECTAR (Non-synonymous Enriched Coding muTation ARchive; http://nectarmutation.org) is a database and web application to annotate disease-related and functionally important amino acids in human proteins. A number of tools are available to facilitate the interpretation of DNA variants identified in diagnostic or research sequencing. These typically identify previous reports of DNA variation at a given genomic location, predict its effects on transcript and protein sequence and may predict downstream functional consequences. Previous reports and functional annotations are typically linked by the genomic location of the variant observed. NECTAR collates disease-causing variants and functionally important amino acid residues from a number of sources. Importantly, rather than simply linking annotations by a shared genomic location, NECTAR annotates variants of interest with details of previously reported variation affecting the same codon. This provides a much richer data set for the interpretation of a novel DNA variant. NECTAR also identifies functionally equivalent amino acid residues in evolutionarily related proteins (paralogues) and, where appropriate, transfers annotations between them. As well as accessing these data through a web interface, users can upload batches of variants in variant call format (VCF) for annotation on-the-fly. The database is freely available to download from the ftp site: ftp://ftp.nectarmutation.org.

## INTRODUCTION

Next-generation sequencing platforms bring a new dimension to genome research by generating ultrafast and high-throughput sequencing data on an unprecedented scale. Important developments including advances in short-read alignment tools (1,2), variation calling software (3), target enrichment strategies (4) and the recent development of desktop-sized sequencing machines (5) have brought large-scale genome sequencing within reach of many more researchers. The challenge in the postgenomic era has therefore shifted from data generation to data interpretation, and, in particular, to linking genotype with phenotype.

**Figure 1.**
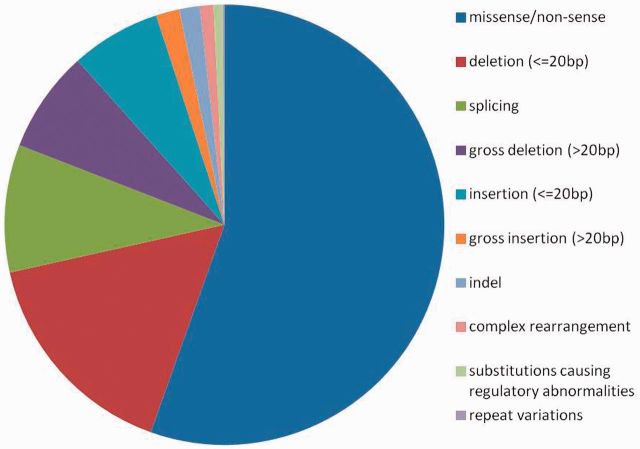
The proportion of HGMD records by their variant types. The data are drawn from the HGMD professional (version 2013.1) where disease-causing mutations are tagged as either ‘DM’ or ‘DM?’, which is defined as ‘pathological mutations reported to be disease causing in the original literature report’. The question mark denotes that a degree of doubt has been found to exist with regard to pathogenicity.

Non-synonymous single nucleotide variants, which cause single amino acid substitutions, are a particular challenge: though most disease-associated variants are non-synonymous SNPs (6), most non-synonymous SNPs are common and appear to be functionally neutral (7). Therefore interpreting the functional importance of novel SNPs is challenging. The majority (54%) of known disease-causing mutations in the Human Gene Mutation Database (HGMD) (8) are missense or nonsense, followed by deletions and splice site variants, which account for 16 and 9%, respectively (see Figure 1).

The same amino acid substitution can be generated by more than one DNA variant because multiple codons encode a single amino acid (codon degeneracy). For instance, variants in the myosin regulatory light chain (MYL2) including c.52T > C (p.Phe18Leu) have been reported to cause familial hypertrophic cardiomyopathy (9). Three alleles at two distinct genomic locations could equally substitute phenylalanine to leucine (c.54C > G, c.54C > A and c.52T > C), although only one of them (c.52T > C) has been previously reported. Other alleles can also substitute this conserved phenylalanine to isoleucine (c.52T > A), valine (c.52T > G), tyrosine (c.53T > A), cysteine (c.53T > G) or serine (c.53T > C). Therefore the phenotype associated with c.52C > T may be relevant in interpreting other alternative missense variants affecting the same codon. There are publicly (or commercially) available databases, which catalogue disease-related variants based on published literature or their own experiments. For example, the Human Genome Variation Society (HGVS) maintains a website (http://www.hgvs.org/dblist/dblist.html) listing Locus-Specific Databases and other disease-related variant databases. However, existing databases based on genomic position do not readily link reported variants to all alternative alleles affecting the same amino acid residue.

Here we introduce NECTAR (Non-synonymous Enriched Coding muTation ARchive), which is a database of non-synonymous variants responsible for disease and altered protein function. NECTAR aids interpretation of missense variants by giving access to existing annotations in two new ways: first, by cross-linking annotations at the relevant codon level and, secondly, by transferring annotations between evolutionarily related proteins. Known disease variants are compiled from publicly available databases and expanded to archive possible alternative non-synonymous alleles at the same codons where the original variants are located. NECTAR also archives possible non-synonymous variants that substitute other functionally annotated amino acid residues. The locations of disease variants and functional residues are propagated across protein paralogues, which enables interrogation at the equivalent positions. NECTAR accepts genetic variants in a variant call format (VCF) file (10), then annotates them on-the-fly. NECTAR is freely available to download via the web (http://nectarmutation.org) and an FTP site (ftp://ftp.nectarmutation.org) where a simple shell script is provided for those who wish to mirror the data locally.

## DATA COLLECTION AND ANNOTATION

### Compiling external resources

**Figure 2.**
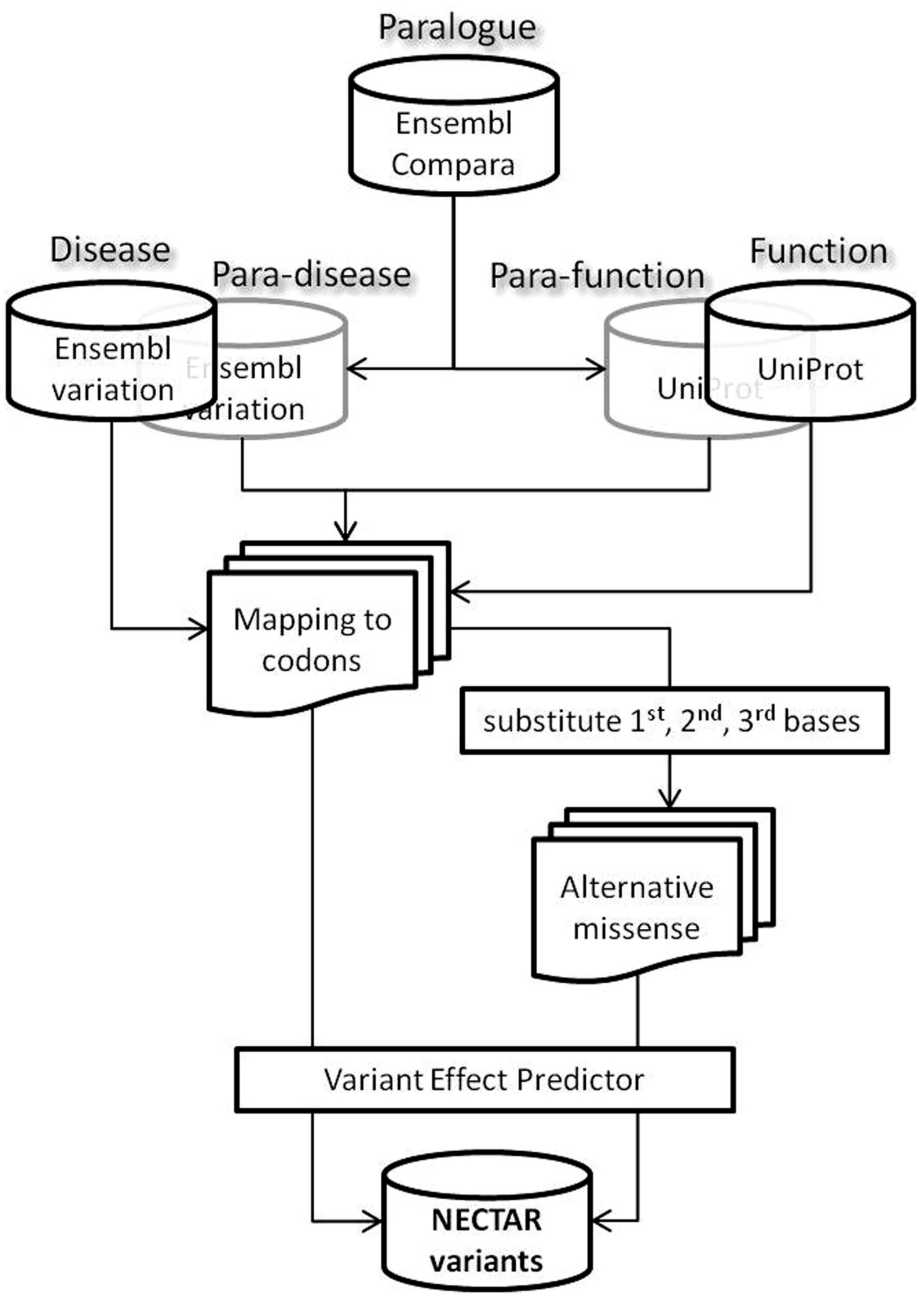
A schematic diagram of NECTAR framework. The Ensembl databases (Core, Variation and Compara) were downloaded and locally mirrored to speed up database queries using their API. UniProt XML files were also mirrored and parsed to construct an equivalent in-house SQL version. MySQL was used for the main back-end database management system and Perl for data processing. See the main text for the description of the workflow.

Figure 2 explains the data collection and annotation pipeline of NECTAR. Non-synonymous disease variants are collated from the Ensembl variation database (11,12) and UniProt human polymorphisms and disease variants (http://www.uniprot.org/docs/humsavar) (13,14). Among the Ensembl variants, only those from (i) Catalogue of Somatic Mutation in Cancer (COSMIC) (15), (ii) pathogenic or probable-pathogenic variants from ClinVar (http://www.ncbi.nlm.nih.gov/clinvar) or (iii) HGMD-public (8) resources were used; their genomic positions were mapped later to their corresponding Ensembl proteins via canonical transcripts (12,16). UniProt variants, of which only disease variants were used, were transferred to their corresponding Ensembl proteins using bl2seq, a pair-wise alignment software tool, of the NCBI-BLAST software package (17). For the definitions of functional amino acid residues, 12 categories of function annotations were chosen from UniProt and their positions were mapped to their corresponding Ensembl human proteins. Table 1 and Table 2 list the external data sources and the number of disease variants and functional amino acids used in NECTAR. To remain up-to-date, NECTAR aims to update dependent data sets as each new Ensembl version is released.

**Table 1.** The source of disease variants and the number of variations in NECTAR

Sources of variants	Number of genes	From the source	NECTAR	
		Number of amino acid substitutions^a^	Number of alternative amino acid substitutions^a^	Number of DNA variants
UniProt^b^	1918	24 730	106 449	145 001
COSMIC^c^	16 794	448 637	2 138 093	2 862 016
HGMD-public^c^	2826	Not available	231 504	315 024
ClinVar^c^	1969	11 724	56 257	74 131

^a^This number is based on the Ensembl proteins translated from the Ensembl canonical transcripts.

^b^Version 2013_08.

^c^As a part of Ensembl variation database version 73.

**Table 2.** Twelve functional annotations from UniProt and the number possible non-synonymous variants in NECTAR

Category of function		Number of genes	UniProt^a^	NECTAR	
Abbreviation	description		Number of amino acids^b^	Number of possible amino acid substitutions^b^	Number of DNA variants
CA_BIND	Position(s) of calcium binding region(s) within the protein	230	6075	38 937	43 994
ZN_FING	Position(s) and type(s) of zinc fingers within the protein	1687	241 415	1 539 638	1 745 301
DNA_BIND	Position and type of a DNA-binding domain	563	47 152	295 359	332 300
NP_BIND	Nucleotide phosphate binding region	1618	28 124	10 560	190 097
ACT_SITE	Amino acid(s) directly involved in the activity of an enzyme	1987	3318	22 704	25 911
METAL	Binding site for a metal ion	1239	5775	39 794	45 463
BINDING	Binding site for any chemical group (coenzyme, prosthetic group, etc.)	1584	4375	28 249	32 042
MOD_RES	Modified residues excluding lipids, glycans and protein cross-links	6954	32 530	195 862	224 744
LIPID	Covalently attached lipid group(s)	614	908	5996	6804
CARBOHYD	Covalently attached glycan group(s)	4152	16 622	115 143	131 637
DISULFID	Cysteine residues participating in disulfide bonds	2894	32 371	226 586	258 954
CROSSLNK	Residues participating in covalent linkage(s) between proteins	445	955	6639	7593

^a^Version 2013_08.

^b^This number is based on the equivalent Ensembl proteins translated from the Ensembl canonical transcripts.

### Enriching annotations

NECTAR compiles possible putative missense variants based on known disease variants and functional amino acids as described above. There are three classes of ‘NECTAR variant’: (i) known disease-related variants and possible alternative missense alleles affecting the same codon, (ii) putative non-synonymous variants substituting functional amino acid residues, (iii) variants annotated by sequence homology (paralogue annotations). The amino acid positions of disease variants and functional residues were transferred and marked to their equivalent positions of their paralogues using the gene paralogy definition adopted from the EnsemblCompara GeneTree (18). They are annotated as ‘Para-disease’ and ‘Para-function’ as shown in Figure 2. Using the TranscriptMapper object of the Ensembl core Application Programming Interface (API), the amino acid positions of disease variants, functional residues and their paralogue annotations were further mapped onto the codon positions of their corresponding Ensembl canonical transcripts. Possible alternative codons were generated by replacing the first, second and third base of the original codon one-by-one and retained those that were non-synonymous. In addition to paralogue annotations, NECTAR provides possible missense variants for manually curated UniProt disease and function annotations that are only reported at amino acid residue level through the UniProt website. The functional effects of NECTAR variants were estimated by SIFT (19) and PolyPhen (20), which are pre-computed by the Variant Effect Predictor (VEP) (21) as part of the Ensembl variation API (11,22).

## FEATURES OF NECTAR

**Figure 3.**
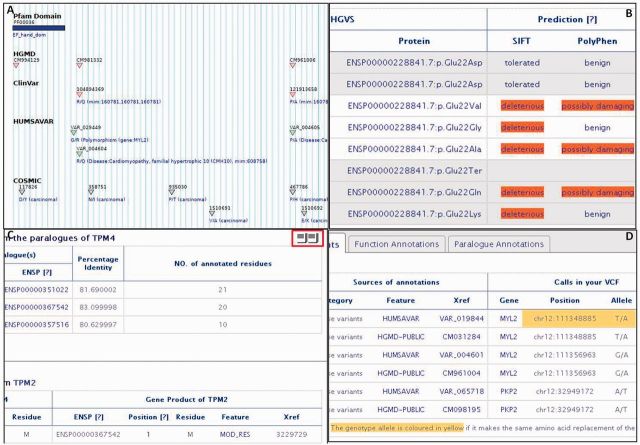
Screen captures of the NECTAR website. (**A**) A GBrowse image shows the locations of disease-related amino acid substitutions and a Pfam domain (coloured in blue bar) along the sequence of MYL2 protein. A fine control of GBrowse image is possible if the image is being clicked. (**B**) One possible nonsense and seven missense variants are displayed at the Glu22 of MYL2 protein where Glu22Lys is originally reported by UniProt (VAR_004603). Their functional effects, predicted by SIFT and PolyPhen, are also shown. (**C**) Paralogue annotations of TPM4 are displayed. FTP links are coloured in red on the upper right corner. (**D**) NECTAR annotations are made on-the-fly from a user-provided VCF input. A variant is coloured in yellow because it makes the same amino acid substitutions as reported from the source (VAR_019844 from UniProt). The results can be downloaded as a spread sheet. The input was from http://nectarmutation.org/main/static/nectar_dummy.vcf.

NECTAR is searchable by gene name (HUGO Gene Nomenclature Committee identifier) or disease name, which then lists known disease-associated genes. The web search is enhanced with the Google Custom Search to facilitate retrieving specialized information in NECTAR. There are four subsections for each gene page: (i) disease associations, (ii) disease variants, (iii) function annotations and (iv) paralogue annotations. Each variant table is accompanied with a visualization of the variant positions [Gbrowse (23)] with functional and protein domain annotations (see Figure 3). NECTAR variants, accessed via the web interface, are provided at the amino acid residue level referenced to the protein translated from the Ensembl canonical transcript (12,16). Each table is also accompanied by FTP links for direct download (see Figure 3C). Each subsection is further explained below except the disease association section, which is from the UniProt general annotation section. NECTAR has been tested on following major web browsers: Internet Explorer 8 and 9, Firefox 3.6.26, 22.0 and 24.0, Chrome 30.0 and Safari on iOS7.

### Disease variants

Table 1 lists the sources of disease variants used in NECTAR and compares the number of variants reported from the source and that of possible alternatives identified in NECTAR. For example, UniProt reports 24 730 amino acid substitutions responsible for diseases. From the same source, NECTAR identifies additional 106 449 alternative substitutions at the reported codon positions by substituting first, second or third bases of the original codons (see Figure 2 for details); all in all, 145 001 variants are available for alternative substitutions together with the original reports from UniProt. NECTAR identifies similar fold of increase in the number of alterative substitutions from COSMIC and ClinVar.

### Function annotations

UniProt provides the most comprehensive catalogue of protein sequences and function annotations (http://www.uniprot.org/manual/sequence_annotation), which describe regions or sites of interest in the protein sequence. NECTAR archives amino acids annotated as functional residues by UniProt and extends to compile possible alternative amino acids at the functional positions together with the genetic variants responsible for the amino acid changes. Table 2 shows 12 functional annotations used in NECTAR and the number of reported amino acids relevant to the functional categories and the number of possible amino acid replacements identified by NECTAR. Like disease variants, the effects of NECTAR functional variants are predicted by the SIFT and the PolyPhen—they are presented either from the NECTAR website if users click the position of the relevant functional residues, or as a FTP link for a batch download from the NECTAR gene page where user queried (see Figure 3).

### Paralogue annotations

Proteins of shared ancestry may exhibit analogous functions, mediated by conserved sequence motifs or 3D structures (24,25). Therefore, it is equally interesting to investigate the effect of non-synonymous variants at the equivalent amino acid positions between close homologues. Sequence homology information is used to annotate uncharacterized genes and proteins (26–29) and for the analysis of non-synonymous SNPs and their relation to disease (30). Recently, we described an approach using paralogue annotations for the functional annotation of non-synonymous variants, first validated in inherited cardiovascular disease (31). A similar approach was adopted in NECTAR to facilitate propagation of disease and function information to uncharacterized proteins. For example, TPM4 (tropomyosin alpha-4 chain), one of four tropomyosin genes, shares >80% protein sequence identity with its paralogues (TPM1, TPM2, and TPM3). There are no reported disease variants for TPM4, at the moment of this writing, either from HGMD-public or UniProt, whereas its paralogue TPM1 is reported to be responsible for familial hypertrophic cardiomyopathy type 3 (MIM:115196) and cardiomyopathy dilated type 1Y (MIM:611878); TPM2 for nemaline myopathy type 4 (MIM:609285) and distal arthrogryposis type 1A (MIM:108120); TPM3 for nemaline myopathy type 1 (MIM:609284) and thyroid papillary carcinoma (MIM:188550). NECTAR annotates 48 amino acids of TPM4 protein where their equivalent alignment positions are annotated as disease-related either by HGMD-public, ClinVar or UniProt from its paralogues (see Figure 3C).

### Annotation on-the-fly

When assessing putative disease-causing variants, e.g. for clinical diagnostics, the first step is to consult databases of known disease variants [e.g. ClinVar, HGMD, T1Dbase and Locus-Specific Databases (http://www.hgvs.org/dblist/glsdb.html)] or public variation data (e.g. dbSNP, COSMIC and SwissVar) to see whether the observed variants have been previously reported and characterized. NECTAR users can upload their variations formatted in a VCF file (10) to have them annotated on-the-fly. This looks up NECTAR variants and annotates non-synonymous variants, if any, from the user input in the three annotation sections (disease, function and paralogue) (see Figure 3D). For those wish to use the Ensembl VEP (21), which predicts the functional consequences of genomic variants, NECTAR runs this locally and provides a link, as shown in Figure 3D, where users can download the result as a spreadsheet. This provides a useful complement to NECTAR, which only annotates missense variants at the moment; the VEP will miss NECTAR annotations instead, if there are any. The online Supporting Information explains technical details of the NECAR web and FTP site including implementation of the local VEP.

## RELATED WORKS

While we are not aware of any web application providing ready access to the range of codon-centric annotations compiled in NECTAR, there are other databases and web servers that could be jointly used to compile annotations equivalent to those provided in NECTAR. A VEP plug-in (https://github.com/ensembl-variation/VEP_plugins) is available that looks for existing variants affecting the same codons as a list of user-provided variants. Also, UCSC Variant Annotation Integrator (http://genome.ucsc.edu/cgi-bin/hgVai), ANNOVAR (32), variant tools (33) and KGGSeq (34) provide a functional prediction and annotation for user provided variants using dbNSFP (35), which is a database of all potential non-synonymous single-nucleotide variants in the human genome. The dbNSFP provides functional prediction scores and conservation scores, which are pre-computed using a number of tools. Also Whole Human Exome Sequence Space (ftp://genetics.bwh.harvard.edu/pph2/whess) archives all putative single-nucleotide non-synonymous (missense) codon changes and provides annotations of pre-computed set of PolyPhen-2 predictions. However, even though it is possible to have codon-centrc annotations with additional efforts (e.g. programming for customized annotations), some of them fail to provide cross-references for known disease variants and UniProt function annotations at the codon level. None of the other web servers/databases provides equivalent sequence positions across paralogous proteins, although this information can be extracted from alternative sources [e.g. Ensembl Compara (18), UCSC Genome Browser MultiZ alignments (36) and eggNOG (37)].

## DISCUSSION

NECTAR allows data mining of genetic variants not only from known disease and function annotations, but also from alternative amino acids (and their responsible genetic alleles) shared at the same codons where current annotations are available, further enhanced to facilitate the transfer of annotations between equivalent residues across protein paralogues. The phenotypic consequences of NECTAR variants can be inferred from linked reports. NECTAR provides access to publically available data that may be usefully applied in both diagnostic and research settings, but these phenotype data are not curated. Users considering a clinical diagnostic application should be sure to independently evaluate the quality of the source data. Also there are a few things to consider when using NECTAR. NECTAR only covers single base substitutions in protein coding regions. As shown in Figure 1, 45% of disease-causing variants are not single base substitutions; NECTAR does not evaluate other variant classes such as radical frame shifts and essential splice site variants. Recent study reveals most somatic mutations have little or no implication for cancer development, with only smaller numbers drive tumour (38); they are not distinguished in NECTAR for mutations from the COSMIC database (15).

## SUPPLEMENTARY DATA

Supplementary Data are available at NAR Online, including [39].

## FUNDING

This research was supported by the Academy of Medical Sciences; the Wellcome Trust; the British Heart Foundation [grant number SP/10/10/28431]; Arthritis Research UK; Fondation Leducq; the NIHR Cardiovascular Biomedical Research Unit at Royal Brompton & Harefield NHS Foundation Trust and Imperial College London. Funding for open access charge: the British Heart Foundation and Wellcome Trust.

*Conflict of interest statement*. None declared.
